# Early African Swine Fever Dynamics in Wild Boar After Reemergence in Catalonia, Spain: Surveillance‐Based Estimates of Initial Spread

**DOI:** 10.1155/tbed/3757757

**Published:** 2026-09-18

**Authors:** Xavier F. Aguilar, Liani Coronado, Lola Pailler-García, Albert Sanz, Carlos Sánchez, Marta Busoms, Francisco Domenes, Àlex Cobos, Enric Vidal, Miquel Nofrarías, Guillermo Cantero, Carme Rosell, Joana Colomer, Francesc Accensi, Joaquim Segalés, Jordi Argilaguet, Natàlia Majó, Carles Vilalta, Osvaldo Fonseca-Rodríguez

**Affiliations:** ^1^ Unitat Mixta d’Investigació IRTA-UAB en Sanitat Animal, Centre de Recerca en Sanitat Animal (CReSA), Campus de la Universitat Autònoma de Barcelona (UAB), Bellaterra 08193, Catalonia, Spain, uab.cat; ^2^ IRTA, Programa de Sanitat Animal, Centre de Recerca en Sanitat Animal (CReSA), Campus de la Universitat Autònoma de Barcelona (UAB), Bellaterra 08193, Catalonia, Spain, uab.cat; ^3^ World Organization for Animal Health (WOAH) Collaborating Centre for the Research and Control of Emerging and Re-Emerging Swine Diseases in Europe (IRTA-CReSA), Barcelona, Spain; ^4^ Departament d’Agricultura, Ramaderia, Pesca i Alimentació de la Generalitat de Catalunya, Barcelona, Catalonia, Spain; ^5^ Departament de Sanitat i Anatomia Animals, Facultat de Veterinària, Universitat Autònoma de Barcelona (UAB), Campus de la UAB, Bellaterra 08193, Spain, uab.cat; ^6^ MINUARTIA, Wildlife Consultancy, Barcelona 08011, Spain; ^7^ Department of Evolutionary Biology, Ecology and Environmental Sciences, Faculty of Biology, University of Barcelona, Barcelona 08007, Spain, ub.edu; ^8^ IRBio, Institut de Recerca de la Biodiversitat, University of Barcelona, Barcelona, Spain, ub.edu

**Keywords:** African swine fever, carcass detection, minimum convex polygon, spatial spread, surveillance, wild boar

## Abstract

African swine fever (ASF) reemerged in free‐ranging wild boar in Catalonia, northeastern Spain, in November 2025, representing the first detection in Spain since 1994. We described the initial outbreak phase using surveillance data collected from 25 November 2025 to 23 February 2026. ASF virus (ASFV) positivity by real‐time quantitative polymerase chain reaction (qPCR) was estimated overall and by surveillance stream; mortality timing was reconstructed probabilistically from estimated death‐date intervals; and early front displacement was analyzed using leading‐edge cumulative minimum convex polygons (MCPs), linear and generalized additive models (GAMs), segmented regression, and directional modeling. Among 1483 wild boars with definitive qPCR results, 201 were ASFV qPCR‐positive, giving an overall qPCR positivity of 13.6% (95% confidence interval [CI] 11.9–15.4). Positivity was substantially higher in enhanced passive than in active surveillance (28.2% vs. 0.9%; positivity ratio [PR] 32.11, 95% CI 15.21–67.78; *p* < 0.001). A probabilistic cumulative mortality curve based on 198 positive carcasses indicated that, by the date of the first detected carcass, expected cumulative mortality had already reached 25.8 deaths (13% of reconstructed total mortality). Leading‐edge analysis estimated an apparent mean radial front‐displacement rate of 0.042 km/day (95% CI 0.029–0.055). The surveillance‐derived distance–time relationship was nonlinear, indicating that the apparent front‐displacement rate varied over the study period, with a segmented‐regression breakpoint at 119 days and higher apparent front‐displacement thereafter (0.106 vs. 0.021 km/day). The exploratory directional analysis indicated the fastest predicted advance toward the South–Southwest sector. These surveillance‐derived estimates describe the observed leading edge under the surveillance and control conditions operating during the study period and should not be interpreted as direct estimates of ASFV transmission speed. These findings support an adaptive, risk‐based response that prioritizes carcass search and removal and incorporates directional spread information to refine surveillance and zoning as the outbreak evolves. The analytical framework, combining reconstruction of estimated dates of death with simple descriptors of early epidemic spread, may support early epidemiological assessment of ASF incursions in other newly affected settings.

## 1. Introduction

African swine fever (ASF) is a highly contagious viral disease of suids that affects both domestic pigs and Eurasian wild boar (*Sus scrofa*) and can cause high mortality rates in naïve populations [1, 2]. The etiological agent, ASF virus (ASFV; species *Asfivirus haemorrhagiae*, genus *Asfivirus*, family Asfarviridae) [3], is a large double‐stranded DNA virus that replicates predominantly in macrophages and shows marked environmental and tissue stability, supporting transmission through contaminated materials and carcasses, features that complicate control in free‐ranging populations [1, 4]. Despite substantial advances in vaccine research, prevention and control still rely on early detection, biosecurity, movement restrictions, rapid removal/management of infectious material, and, in the case of domestic pig holdings, stamping out [2].

ASF has evolved into a major transboundary animal health threat. According to the World Organization for Animal Health (WOAH) through the World Animal Health Information System (WAHIS), ASF has been reported across multiple world regions and numerous countries in recent years, affecting both domestic pigs and wild boar [5–7]. The contemporary pandemic is largely driven by ASFV genotype II, introduced into the Caucasus in 2007 and subsequently expanding widely in Europe, Asia, Oceania, and occasionally in the Caribbean [2, 6, 8].

Following the 2007 introduction in Georgia, genotype II ASFV spread across multiple European countries over subsequent years, affecting domestic pigs and/or wild boar. Repeated incursions and re‐/emergence events reinforce the need for sensitive surveillance and rapid delimitation [8–11]. In Europe, ASF epidemiology is strongly influenced by the wild boar–habitat cycle, in which carcasses and environmental contamination contribute to local persistence and contiguous spatial spread [4]. Despite extensive control efforts, ASF continues to spread across connected wild boar populations, particularly in eastern and central Europe. Control in free‐ranging wild boar populations remains challenging due to their increasing abundance and the difficulty of reversing this trend [12, 13]. Long‐distance ASFV introductions, occurring far from the nearest infected wild boar populations, have also been reported in Belgium [14], Sweden [15], mainland Italy [16], and the Czech Republic [17], and are therefore unlikely to reflect contiguous wild‐boar‐mediated spread. These events underline the important role of human‐mediated activities in ASFV long‐distance spread [18]. This spread is facilitated by the high environmental stability of the virus, including in pork and meat products [19] and by the exploitation of anthropogenic waste and food resources by wild boar [20, 21]. Control of such foci relies on rapid response, including early detection, delimitation of affected areas, and immediate implementation of measures such as zoning with physical barriers, management of wild boar densities, and removal of carcasses and contaminated materials [18].

Spain eradicated ASF (caused by ASFV genotype I) from the domestic pig population in 1995 after prolonged control efforts [22]. In November 2025, in Catalonia, the first ASFV‐positive wild boars were associated with two carcasses detected in the Cerdanyola del Vallès municipality. The competent veterinary services responsible for passive wildlife surveillance in Catalonia were notified of the carcasses on 25 and 26 November 2025, and ASF was officially confirmed by the national reference laboratory on 27 November 2025 [23–25]. This represented the first ASF detection in Spain since November 1994 [26], and the initial response included the establishment of operational zones and intensified surveillance in the affected area [24].

Quantitative descriptions of the earliest phase of ASF incursion in newly affected regions remain limited despite their importance for targeting surveillance and early containment. Important knowledge gaps remain regarding surveillance yield, the timing and progression of mortality in relation to estimated dates of death, and the initial pace and direction of spatial expansion. These early processes are epidemiologically and operationally important because they shape outbreak delimitation, carcass‐search prioritization, and the design of adaptive control measures during the first months of response. In this context, the reemergence of ASF in wild boar in Catalonia generated early quantitative evidence from a newly affected setting in southwestern Europe. Therefore, the objective of this study was to describe the initial 3‐month phase of ASF in wild boar in Catalonia using enhanced surveillance data collected between 25 November 2025 and 23 February 2026. Specifically, we (i) described overall ASFV positivity by surveillance stream (active versus enhanced passive surveillance), (ii) described the spatial distribution of tested and ASFV‐positive wild boar in relation to operational risk zones, (iii) reconstructed cumulative mortality while accounting for uncertainty in estimated dates of death, and (iv) characterized early front displacement and directional heterogeneity in spread during the study period.

## 2. Materials and Methods

### 2.1. Study Design and Operational Risk Zoning

A descriptive observational analysis of ASF surveillance data in free‐ranging wild boar (*S. scrofa*) in Catalonia (northeast Spain) was performed. The analytic period began on 25 November 2025, corresponding to the first field detection date of an ASFV quantitative polymerase chain reaction (qPCR)‐positive carcass, and included samples collected up to 23 February 2026. The outbreak was ongoing at the time of the analysis.

To characterize the response setting and support descriptive mapping of the initial phase, the competent veterinary authority initially defined operational risk zones as concentric buffers around the index cases, the first two detected ASFV‐positive carcasses. A 6‐km “high‐risk” zone and a 20‐km “buffer/low‐risk” zone, with buffers merged across the two initial detections, were implemented. This zoning approach reflects guidance emphasizing intensified carcass search and surveillance around detected cases during early ASF management in wild boar [17].

The high‐risk zone includes part of the metropolitan area of Barcelona and extends to the north of it. It is a densely populated landscape characterized by a high density of transport infrastructure, including roads, highways, and railways. Urban areas are interspersed with patches of shrublands, agricultural land, Mediterranean forests (predominantly *Pinus halepensis* and *Quercus ilex*), and riparian vegetation in watercourses that serve as ecological corridors [20]. The Collserola massif, which also constitutes a natural park (CNP), represents the largest continuous forested area within this landscape. The area supports moderate to high densities of wild boar, estimated at ~5–10 individuals per km^2^ [27]. Wild boar densities in such Mediterranean areas show strong interannual variation and are strongly influenced by climate factors affecting food availability [28]. These estimates were derived from driven hunt data collected during the hunting season and adjusted for hunting effectiveness. They should be interpreted cautiously as approximate hunting‐based estimates rather than as complete estimates for the entire outbreak area, particularly because monitoring in Collserola covered a limited area and may underrepresent synanthropic wild boars not exposed to hunting. Wild boar populations in the area comprise individuals exhibiting varying degrees of habituation to humans and differing levels of reliance on anthropogenic food resources [21, 29].

Once the first cases were detected, containment measures in line with international recommendations were implemented, including the delimitation of the infected zone, restrictions on public access to infected areas, on hunting, the installation of fencing, and active searches for carcasses using trained dogs [30, 31]. Physical barriers along transport infrastructure were immediately installed using major roads and railways, and crossing structures such as drainage systems and underpasses were reinforced to restrict wild boar movements from the affected area. Fences or reinforcements were installed following the prescriptions for wild boar‐proof fencing [32]. These barriers were implemented to limit the northward, eastward, and westward spread of the epidemic into regions with larger and more connected forest habitats supporting higher densities of wild boars [27].

Wild boar carcasses and remains were actively searched by rangers and police using trained dogs, which were critical to improving detection success in the rugged, densely vegetated terrain. Because the affected area also included urban areas and high density of roads, a portion of wild boar carcasses was also reported by citizens and by road maintenance personnel in connection with traffic collisions. Wild boars were also captured using techniques that minimized dispersal, such as trapping, thereby reducing the population density and supporting active surveillance in affected areas.

### 2.2. Data and Data Sources

Individual wild boar ASF surveillance records were obtained from the competent authority and included the sampling context, relevant dates, municipality, geolocation, and laboratory results. Records were included in the descriptive analyses when they had a valid ASFV qPCR outcome (positive or negative) and the metadata needed for stratification (surveillance stream and/or operational zone). Records also included the carcass/sample detection category, which was used to derive the surveillance stream grouping applied in the positivity analysis, as described in Section 2.3. For spatiotemporal front analyses, the dataset was further restricted to ASFV qPCR‐positive records with valid projected coordinates and an available reference date of death, as described in Sections 2.4 and 2.5.

ASF status was determined by detection of the ASFV genome by qPCR, following the WOAH‐recommended protocol described by Fernández‐Pinero et al. [33]. For carcasses, pathologists from the Centre for Research in Animal Health (IRTA‐CReSA) estimated a plausible death‐date interval based on postmortem assessment, using an approach consistent with that described by Chenais et al. [15] (see Table S1); when a single event time was required, the midpoint of this interval was used as the reference date of death. Coordinates were transformed to a projected coordinate reference system (ETRS89 /UTM zone 31N; EPSG:25831) to support metric buffering and planar distance calculations, and geospatial processing followed the simple‐features framework [34]. Administrative layers used for cartography were obtained from the Cartographic and Geological Institute of Catalonia.

### 2.3. qPCR Positivity

Overall qPCR positivity was defined as the proportion of ASFV‐positive wild boar among animals with a definitive qPCR result (positive or negative). Positivity was estimated globally and stratified in two ways: (i) by surveillance stream and (ii) descriptively by detection category. For the surveillance‐stream comparison, records classified as captured (net and cage traps, shot in transects, or captured in urban areas posing risk to the public) were grouped as active surveillance because these samples resulted from planned capture or removal activities. All remaining detection categories were grouped as enhanced passive surveillance (whole carcasses found in the field, remains found in the field, traffic collisions, and animals shot on disease suspicion or abnormal behavior). This classification is consistent with the ASF surveillance framework commonly used in Europe, in which structured searches for wild boar carcasses are considered enhanced passive surveillance because they target dead animals rather than actively sampling live populations [35, 36]. Traffic collision records were included in this group because they involved opportunistic detection of dead or injured animals; however, detection categories were also analyzed separately because collision‐derived samples are epidemiologically distinct from carcasses or remains found in the field.

For each proportion, 95% confidence intervals (CIs) were calculated using the Wilson score method [37]. To compare qPCR positivity between active and enhanced passive surveillance, we constructed a 2 × 2 contingency table and estimated the positivity ratio (PR) with its 95% CI calculated using the log‐scale Katz method [38]. Statistical association in the 2 × 2 table was assessed using Pearson’s chi‐square test. Because sampling was surveillance‐based rather than population‐based, these positivity estimates were interpreted as surveillance test positivity rather than population prevalence. Similarly, the PR and Pearson’s chi‐square test were used to quantify the observed contrast within this surveillance dataset and were not interpreted as population‐level comparisons between groups sampled through equivalent processes.

### 2.4. Probabilistic Cumulative Mortality Curve

For ASFV‐positive carcasses, pathologists provided the earliest and latest plausible dates of death. To reconstruct cumulative mortality while accounting for uncertainty in the date of death, we applied an approach analogous to that used for the first ASF outbreak in Sweden [15].

For each carcass, the date of death was assumed to be equally likely on any day within the estimated interval, treating both endpoints as inclusive. Expected daily mortality was obtained by summing all daily probabilities across carcasses, and expected cumulative mortality was calculated as the cumulative sum of the resulting daily values. For visualization, carcasses were ordered according to their estimated death timing, and their field detection dates were overlaid on the cumulative plot. The detection delay was summarized as the difference, in days, between the field detection date and the midpoint of the estimated death interval.

To assess the robustness of the reconstructed cumulative mortality curve to the assumption of a uniform probability of death within each estimated postmortem interval, we performed a Monte Carlo sensitivity analysis using alternative within‐interval distributions. The analysis included a primary uniform scenario corresponding to the same within‐interval assumption used in the main probabilistic reconstruction and six alternative scenarios. These comprised triangular and beta‐PERT distributions with their modes located at 25%, 50%, or 75% of the interval between the earliest and latest plausible dates of death, representing early‐, midpoint‐, and late‐mode assumptions, respectively. For each carcass, one date of death was sampled within its inclusive estimated interval in each of 10,000 Monte Carlo iterations. For each iteration and scenario, we calculated cumulative mortality by the date of first detection, 25 November 2025, the corresponding proportion of the 198 carcasses included in the mortality reconstruction, and the dates on which cumulative mortality reached 25%, 50%, 75%, and 90%. Scenario‐specific results were summarized using medians and 95% simulation intervals, defined by the 2.5th and 97.5th percentiles of the simulated values.

### 2.5. Leading‐Edge Reconstruction Using Cumulative Minimum Convex Polygons (MCPs)

To describe early geographic expansion, we reconstructed a cumulative outbreak envelope using MCPs. MCP/convex hull estimators provide a standard geometric boundary enclosing observed locations and are widely used to summarize the outer extent of a point set [36, 39, 40]. Analyses were restricted to ASFV qPCR‐positive records with nonmissing projected coordinates and an available reference date of death.

Primary cases were defined as the six ASFV‐positive wild boars with the earliest estimated times of death based on death‐date interval assessments. These six cases were selected because their estimated death‐date intervals substantially overlapped, making them compatible with the earliest reconstructed mortality phase. The centroid of this early cluster was used to reduce the influence of any single carcass, given the uncertainty in postmortem interval estimation and carcass detection. Primary cases do not necessarily correspond to the index cases, defined as the first two ASFV‐positive carcasses detected in the outbreak. The spatial reference origin, used as an operational reference for radial displacement analyses, *O*, was defined as the centroid point of the six primary cases and should not be interpreted as the true point of ASFV introduction. The temporal origin *t*_0_ was defined as the earliest reference date of death among these primary cases, where the reference date corresponded to the midpoint of the estimated death interval. In the final dataset, this corresponded to 8 September 2025, based on the oldest primary case.

For each ASFV qPCR‐positive record, we calculated its radial distance from the spatial reference origin, *O*, and the number of days since the temporal origin. For each day, we constructed a cumulative MCP using all ASFV‐positive records with a reference date of death up to that day. To focus on outward expansion rather than interior accumulation of detections, a point observed on a given day was classified as a leading‐edge point only if it was a vertex of the cumulative hull lying outside the previous cumulative hull and thus contributing to the expansion of the cumulative hull area relative to the previous day. This leading‐edge subset was then used for subsequent analyses of overall front displacement, nonlinear temporal change, breakpoint detection, and directional spread.

To evaluate the influence of the spatial‐origin definition, we performed a sensitivity analysis using alternative spatial origins: the earliest single reconstructed death, the centroid of the first two detected carcasses, the centroid of the three earliest reconstructed deaths, the centroid of the five earliest reconstructed deaths, and the centroid of the 10 earliest reconstructed deaths. For each origin definition, we repeated the leading‐edge reconstruction and recalculated the linear apparent front‐displacement rate, segmented regression estimates, and directional spread model. The temporal origin was kept constant across scenarios to isolate the effect of the spatial‐origin definition.

To assess the influence of individual leading‐edge observations on the MCP‐based reconstruction, we performed a leave‐one‐out sensitivity analysis. Each of the 43 observations classified as a leading‐edge vertex in the primary analysis was omitted in turn from the complete ASFV‐positive case dataset. After each omission, the cumulative MCP reconstruction and the linear, segmented, and directional models were repeated using all remaining cases while holding the primary spatial and temporal origins constant. Results were summarized as minimum–maximum ranges across the 43 iterations and were interpreted as sensitivity ranges rather than CIs.

### 2.6. Overall Apparent Front Displacement Over Time

The overall front displacement was analyzed using only the leading‐edge MCP vertices defined in Section 2.5. For each selected observation *i*, the response variable was radial distance from the spatial reference origin 𝑂, defined as dist_*i*_ (km), and the predictor was time since the temporal origin, days_*i*_ (days since *t*_0_). These leading‐edge observations were analyzed sequentially to obtain a summary estimate of mean apparent front displacement, assess whether the leading‐edge distance–time relationship was nonlinear, formally test for a breakpoint in the distance–time relationship, and then examine the directional variation in displacement. Because the analysis was based on surveillance‐detected ASFV‐positive wild boar, the resulting slopes were interpreted as apparent front‐displacement rates of the observed leading edge.

#### 2.6.1. Linear Estimation of Mean Apparent Front‐Displacement Rate

To obtain a summary estimate of the average radial apparent front‐displacement rate over the study period, we fitted an ordinary least squares linear regression model:

disti=β0+β1daysi+εi,

where *β*_0_ is the intercept, corresponding to the expected distance at days_*i*_  =  0, *β*_1_ is the mean apparent radial displacement rate (km/day), and *ε*_*i*_ is the residual error term for observation *i*.

#### 2.6.2. Nonlinear Modeling of Apparent Front Displacement Over Time

To assess whether the distance–time relationship departed from the constant‐rate assumption of the linear model, we then fitted a generalized additive model (GAM) with Gaussian errors and a thin‐plate regression spline for time, using restricted maximum likelihood (REML) for smoothing selection [41, 42]:

disti=α+fdaysi+εi,

where *α* is the intercept, *f*(⋅) is a smooth function of time, and *ε*_*i*_ is the residual error term for observation *i*. Time‐varying apparent front‐displacement rate was obtained from the first derivative of the fitted smooth and expressed as the instantaneous rate of change in the observed radial leading‐edge distance (km/day). As an exploratory aid for subsequent breakpoint analysis, we defined a candidate change day as the value of time at which the absolute second derivative of the fitted smooth was maximal, corresponding to the point of greatest local curvature in the fitted trajectory, thus providing a data‐driven indication of when a formal change‐in‐slope model might be warranted.

#### 2.6.3. Breakpoint Detection Using Segmented Regression

We then used segmented regression to test formally whether the temporal pattern suggested by the GAM was compatible with two linear phases separated by a breakpoint. To evaluate whether the distance–time relationship exhibited a change in slope, we first applied Davies’ test [43], which assesses the null hypothesis of no slope change against the alternative of a slope change at an unknown breakpoint. We then fitted a one‐breakpoint segmented linear regression using Muggeo’s iterative algorithm [44].

The segmented model was parameterized as follows:

disti=β0+β1daysi+β2daysi−ψ++εi,

where *ψ* is the estimated breakpoint (days since *t*_0_), (*x*)_+_denotes the positive‐part function, and *ε*_*i*_ is the residual error term for observation *i*. Under this parameterization, the slope before the breakpoint is *β*_1_, and the slope after the breakpoint is *β*_1_ + *β*_2_, both interpreted as apparent front‐displacement rates (km/day).

To improve numerical robustness, the segmented model was initialized from multiple candidate breakpoint values spanning the central empirical range of days_*i*_, specifically the 0.20, 0.30, 0.40, 0.50, 0.60, 0.70, and 0.80 quantiles of the observed distribution. The converged model with the lowest Akaike information criterion (AIC) was retained. Breakpoint CIs were obtained from the fitted segmented model, and segment‐specific slopes were extracted from the final fit; approximate 95% CIs for slopes were calculated from the corresponding standard errors, assuming asymptotic normality. Model fit was compared with that of the single‐slope linear regression using the AIC and an approximate likelihood ratio test.

#### 2.6.4. Directional Spread (Anisotropy) of the Front

After characterizing the overall temporal pattern of front displacement, we examined whether displacement rates also varied by direction in order to identify sectors of faster or slower apparent spread. Directional variation in front displacement was assessed using only the leading‐edge MCP vertices identified in Section 2.5, consistent with regression‐based approaches proposed to quantify directional spread during the early stages of ASF outbreaks [36]. For each leading‐edge point, we calculated the bearing angle from the spatial reference origin (0° = north, 90° = east; angles wrapped to [0, 360)), the radial distance from the spatial reference origin, and the number of days since the temporal origin.

Directional anisotropy was then modeled using a single GAM fitted to all leading‐edge vertices:

disti=aθi+bθidaysc,i+εi,

where *a*(*θ*) is a direction‐specific intercept function, *b*(*θ*) is a direction‐specific front‐displacement rate (km/day), and *ε*_*i*_ is the residual error term for observation *i*. Both functions were represented using cyclic smooth terms to ensure continuity between 0° and 360°, and smoothing parameters were estimated by REML [42, 45].

The directional front‐displacement rate was obtained as the model‐predicted 1‐day change in radial distance at each angle, evaluated on a 1° grid from 0° to 360°. Pointwise 95% CIs were derived from the fitted model covariance matrix. Because some directions were weakly supported by leading‐edge vertices, angular support rules were used only for prediction masking and graphical display and not for model fitting. Candidate GAM specifications were compared using angular coverage, effective support width, median CI width, curve roughness, in‐sample fit, five‐fold cross‐validated RMSE, and AIC. This comparison included alternative angular support windows and support thresholds used for prediction masking and served as a sensitivity assessment of the directional modeling procedure. The final anisotropy model was selected to balance directional resolution, precision, smoothness, and predictive performance, and predictions were masked where angular support was insufficient.

### 2.7. Software

All analyses were conducted in R Version 4.5.0 [46]. Spatial processing and mapping were performed using sf Version 1.1.0 [34]; GAMs were fitted using mgcv Version 1.9.4 [42]; segmented regression was implemented using segmented Version 2.2.1 [44]; and the tidyverse Version 2.0.0 [47] was used for data management and visualization.

## 3. Results

As of 23 February 2026, the ASF incursion into wild boar was ongoing. The results below represent a right‐censored snapshot of the initial phase.

### 3.1. Spatial Distribution and Operational Risk Zones

**Figure 1:**
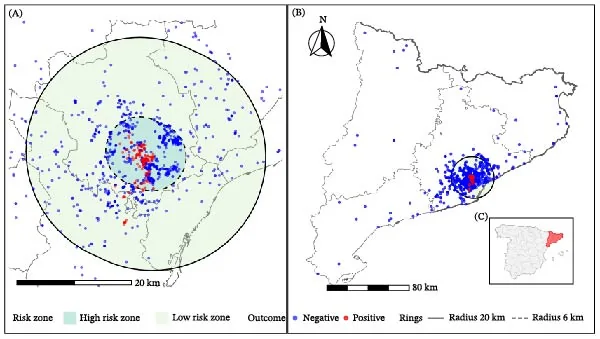
Spatial distribution of ASFV‐tested wild boar in Catalonia from 25 November 2025 to 23 February 2026, and operational risk zones. (A) Close‐up of the outbreak area. (B) Location of the outbreak area within Catalonia. (C) Location of Catalonia within Spain (red area). Tested wild boar are shown as ASFV‐positive (red) and ASFV‐negative (blue). Subfigure (A) shows the operational 6‐km high‐risk zone and 20‐km buffer zone established around the index cases, defined as the first two ASFV‐positive wild boar detected.

The geographic distribution of tested wild boar and ASFV‐positive detections during the study period is shown in Figure 1. Sampling was concentrated in and around the initial outbreak focus, and ASFV‐positive animals were observed predominantly within or near the operational 6‐km high‐risk zone and the surrounding 20‐km buffer zone (Figure 1A). All ASFV‐positive cases were detected within two operational sectors, the core area and a southern sector encompassing the CNP, that were delimited by physical barriers.

### 3.2. Surveillance Data and ASFV qPCR Positivity

Between 25 November 2025 and 23 February 2026, a total of 1483 wild boar with a definitive qPCR result were included in the positivity analysis, of which 201 were ASFV qPCR‐positive, corresponding to an overall qPCR positivity of 13.6% (95% CI 11.9–15.4) (Table 1). Of these, 796/1483 (53.7%) were classified as active surveillance and 687/1483 (46.3%) as enhanced passive surveillance. The qPCR positivity was markedly lower in active surveillance (0.9%, 95% CI 0.4–1.8) than in enhanced passive surveillance (28.2%, 95% CI 25.0–31.7). As a descriptive measure of surveillance yield, test positivity in enhanced passive surveillance was 32.11 times that in active surveillance (PR 32.11, 95% CI 15.21–67.78; *p* < 0.001) (Table 1).

**Table 1:** African swine fever virus qPCR positivity, with 95% confidence intervals, in wild boar by surveillance stream.

Category	Positive	Negative	Total (*n*)	Positivity (95% CI)	PR (95% CI)	*p*‐Value
Global	201	1282	1483	13.6% (11.9–15.4)	—	—
Active surveillance	7	789	796	0.9% (0.4–1.8)	Reference	—
Enhanced passive surveillance	194	493	687	28.2% (25.0–31.7)	32.11 (15.21–67.78)	<0.001

*Note*: PR (positivity ratio) compares enhanced passive surveillance vs. active surveillance. *p*‐Value from Pearson’s chi‐square test.

When detection categories were examined in greater detail, the highest qPCR positivity was observed in carcasses and remains found in the field (46.8%, 95% CI 42.0–51.8), whereas positivity was much lower among animals collected from collisions (1.6%, 95% CI 0.6–4.0) and captured through active surveillance (0.9%, 95% CI 0.4–1.8). Animals shot on suspicion of disease (or abnormal behavior) showed an intermediate qPCR positivity of 13.2% (95% CI: 5.8–27.3). These category‐specific descriptive results are provided in the Supporting Information (Table S2). More detailed information regarding the distribution of ASFV‐positive and ‐negative wild boar across surveillance streams within each operational zone is shown in Figure S1, which illustrates that positivity was concentrated in the high‐risk zone, particularly among carcasses and remains found in the field.

### 3.3. Cumulative ASF Mortality Accounting for Uncertainty in Date of Death

**Figure 2:**
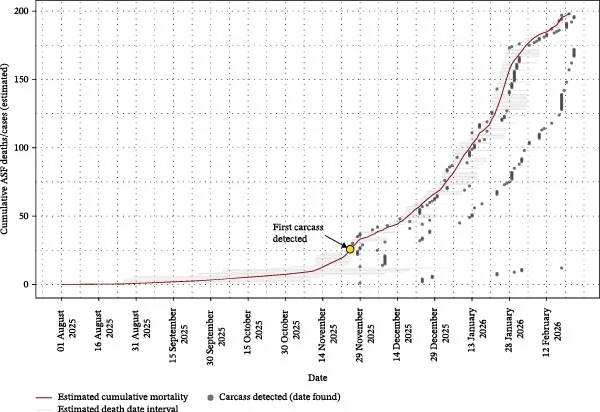
Cumulative ASF mortality with uncertainty in estimated date of death. Horizontal segments represent the estimated death‐date interval for each ASFV‐positive carcass; points indicate the detection date. The red curve shows the expected cumulative mortality over time, computed by assuming a uniform daily probability of death within each carcass’ estimated death interval and summing probabilities across carcasses [15].

A probabilistic cumulative mortality curve was reconstructed using the estimated death‐date intervals for 198 ASFV qPCR‐positive carcasses with valid interval information and detection dates up to 23 February 2026 (Figure 2). The earliest estimated death interval started on 1 August 2025, and the expected cumulative number of deaths increased gradually through autumn before rising more steeply from mid‐December onward. By the date of the first detected carcass in the dataset (25 November 2025), the expected cumulative mortality had already reached 25.8 deaths, equivalent to ~13.0% of the total reconstructed mortality.

The expected cumulative curve reached ~25%, 50%, 75%, and 90% of the total by 18 December 2025, 12 January 2026, 27 January 2026, and 6 February 2026, respectively. The maximum expected daily mortality, corresponding to the steepest increase in the reconstructed curve, occurred on 26 January 2026, with 6.4 expected deaths/day. Detection generally lagging behind the estimated timing of death (Figure 2). The median delay between the midpoint of the estimated death interval and the detection date was 4 days (IQR 4–21), with a mean of 14.5 days (SD 19.1) and a maximum delay of 82 days.

The sensitivity analysis indicated that the reconstructed cumulative mortality curve was broadly robust to alternative assumptions regarding the distribution of the date of death within each estimated interval. Under the primary uniform Monte Carlo scenario, the median cumulative number of deaths by the date of first detection was 26 deaths (95% simulation interval 22–29), corresponding to 13.1% of reconstructed mortality (95% simulation interval 11.1%–14.6%). These simulated estimates closely matched the expected values obtained from the main probabilistic reconstruction, 25.8 deaths and 13.0%, respectively. Across the alternative triangular and beta‐PERT scenarios, the median cumulative number of deaths by the date of first detection ranged from 23 to 29 deaths, corresponding to 11.6%–14.6% of reconstructed mortality. The lowest median estimate was obtained under the beta‐PERT late‐mode scenario, whereas the highest median estimate was obtained under the beta‐PERT early‐mode scenario. The median dates on which cumulative mortality reached 25%, 50%, 75%, and 90% were also similar across assumptions, ranging from 18 to 20 December 2025, 10–13 January 2026, 26–28 January 2026, and 4–6 February 2026, respectively. Scenario‐specific medians and 95% simulation intervals are provided in Table S3, and the reconstructed cumulative mortality trajectories are shown in Figure S3.

### 3.4. Leading‐Edge Reconstruction and Overall Apparent Front Displacement

**Figure 3:**
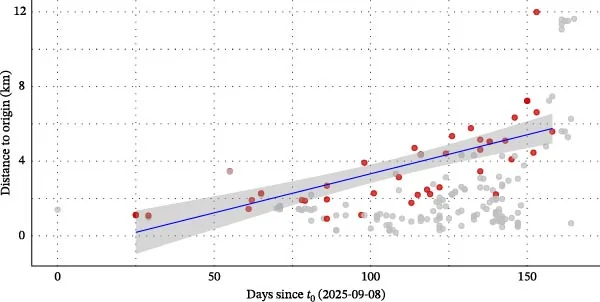
Radial distance from the defined spatial reference origin versus time since *t*_0_ for ASFV qPCR‐positive cases, with an ordinary least squares linear fit. Leading‐edge MCP vertices used for front estimation are shown in red and other ASFV‐positive detections in gray; the blue line shows the fitted regression with 95% confidence band.

**Figure 4:**
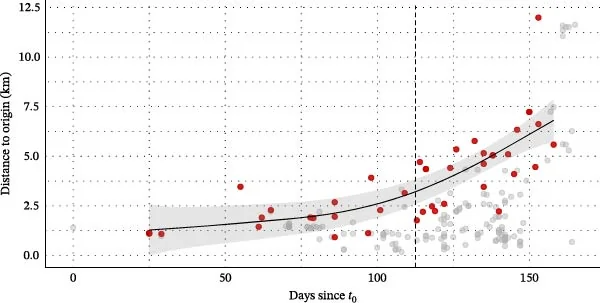
Radial distance from the defined spatial reference origin versus time since *t*_0_ for ASFV qPCR‐positive cases, with a generalized additive model (GAM). Leading‐edge MCP vertices are shown in red and other ASFV‐positive detections in gray. The black curve shows the fitted smooth with a 95% confidence band. The vertical dashed line indicates an exploratory candidate change day, defined as the time point with the maximum absolute second derivative (maximum curvature) of the fitted smooth.

**Figure 5:**
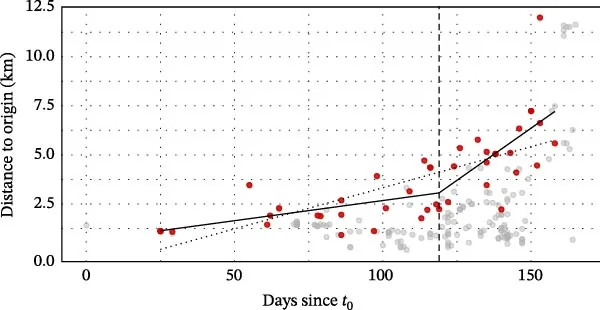
Segmented (piecewise) regression of radial distance against time since the defined temporal origin (*t*_0_). Gray points represent all ASFV‐positive cases and red points indicate the leading‐edge vertices used for model fitting. The solid line shows the fitted segmented model; the dotted line shows the fitted single‐slope linear model for comparison. The vertical dashed line indicates the estimated breakpoint (*ψ*).

**Figure 6:**
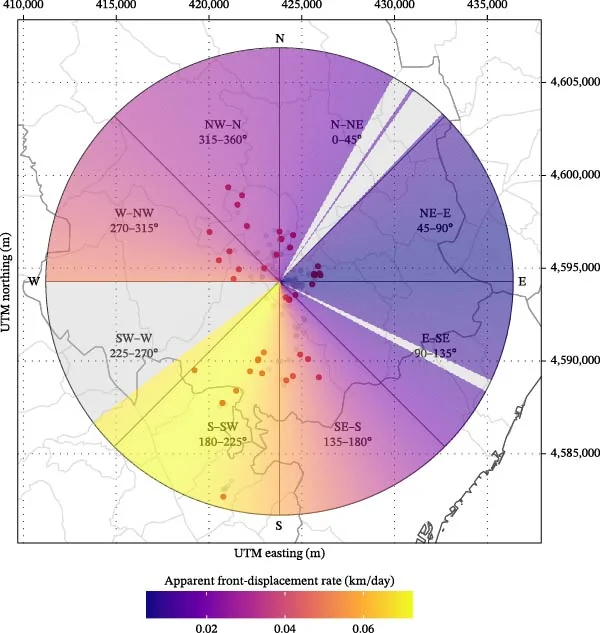
Directional variation in estimated apparent front‐displacement rate (anisotropy). Colored sectors show smoothed direction‐specific front‐displacement rate by bearing from the defined outbreak‐origin; gray sectors indicate directions masked due to insufficient angular support (fewer than five leading‐edge observations within ±35° of the sector bearing). Leading‐edge vertices are shown in red, and other ASFV qPCR‐positive cases in gray.

Using the centroid of the six ASFV‐positive wild boars with the earliest estimated time of death as the spatial reference origin and 8 September 2025 as the temporal origin (*t*_0_), 43 observations were identified as leading‐edge vertices contributing to outward expansion of the cumulative MCP and were retained for front‐displacement modeling (Figures 3–6).

In the linear regression model fitted to this subset (Figure 3), radial distance from the defined spatial reference origin increased with time at an estimated apparent mean front‐displacement rate of 0.0418 km/day (95% CI 0.0292–0.0545). Over the analyzed period, the observed leading edge reached a maximum radial displacement of ~12 km from the defined spatial reference origin.

In the spatial origin sensitivity analysis, the overall linear apparent front‐displacement rate was relatively stable across alternative origin definitions, with estimates ranging from 0.0415 to 0.0474 km/day (Table S4).

All 43 leave‐one‐out iterations were successfully completed. The final cumulative MCP area, maximum radial displacement, and overall linear apparent front‐displacement rate were also relatively stable across iterations (Table S6).

#### 3.4.1. Nonlinear Evolution of Observed Front Displacement

The GAM showed a nonlinear increase in the observed leading‐edge distance over time (edf = 2.49, *F* = 7.16, *p* < 0.001), with 62.9% deviance explained (adjusted *R* ^2^ = 0.605; *n* = 43) (Figure 4). The fitted smooth indicated a modest increase in the observed front displacement early in the study period, followed by a steeper increase later. An exploratory candidate change day was identified at 112.6 days since *t*_0_ (approximately late December 2025), corresponding to the point of maximum absolute curvature of the fitted smooth, and was used as a visual guide motivating formal change‐in‐trend modeling.

#### 3.4.2. Breakpoint and Change in Spread Rate

Consistent with the nonlinearity identified by the GAM, Davies’ test supported a change in slope in the observed distance–time relationship (*p* = 0.0126). In the segmented regression, the estimated breakpoint occurred at 119.0 days (95% CI: 98.4–139.6). The estimated apparent front‐displacement rate increased from 0.021 km/day (95% CI 0.003–0.038) before the breakpoint to 0.106 km/day (95% CI 0.045–0.168) after the breakpoint. Compared with the single‐slope linear regression, the segmented model provided a better statistical fit (likelihood ratio test = 11.421, df = 2, *p* = 0.0033).

Segmented regression estimates were more sensitive to the spatial‐origin definition than the overall linear estimate. The breakpoint in the primary analysis remained close to the primary estimate when the spatial origin was defined as the centroid of the first two detected carcasses or of the three or five earliest reconstructed deaths but shifted to ~136 days when the origin was defined as the location of the earliest reconstructed death or the centroid of the 10 earliest reconstructed deaths. Postbreakpoint apparent front‐displacement rates ranged from 0.1023 to 0.2478 km/day across scenarios (Table S4).

Segmented regression estimates also showed greater variability across the leave‐one‐out iterations, although the postbreakpoint rate remained higher than the prebreakpoint rate in every iteration (Table S6).

#### 3.4.3. Directional Spread (Anisotropy) of the Front

The exploratory directional analysis indicated variation in apparent front‐displacement rates among directions, with the highest predicted rate toward the south–southwest sector (Figure 6 and Figure S2). In the selected directional GAM, both the angular smooth term and the angular‐by‐time smooth term were significant (edf = 3.45, *p* = 0.005; edf = 3.97, *p* < 0.001), indicating directional variation in apparent front displacement and its temporal evolution. The model explained 78.7% of the deviance (adjusted *R* ^2^ = 0.741). Across angles with sufficient data support, the predicted apparent directional displacement rate ranged from 0.0048 km/day at 83° to 0.0721 km/day at 213°. Among supported angles, displacement rates were lowest in the NE–E sector, with a mean predicted rate of 0.0065 km/day over 45°–90°, remained low in the E–SE sector (mean 0.0100 km/day), increased through the SE–S sector (mean 0.0371 km/day), and reached their highest values in the S–SW sector (mean 0.0684 km/day), with the maximum at 213°. Although estimates for supported angles from 225° to 233° also remained high (0.0681–0.0705 km/day), most of the SW–W sector was masked because of insufficient angular support. Masked angular intervals occurred at 29°–33°, 36°–43°, 115°–118°, and 234°–270°.

Directional predictions were more consistent across spatial‐origin definitions. The fastest predicted direction remained within the S–SW sector across all origin definitions, with maximum predicted bearings ranging from 210° to 216°. The fastest predicted direction was also relatively stable across leave‐one‐out iterations (Table S6). Directional support was uneven across angular sectors, with some sectors represented by few or no leading‐edge observations. Predictions in sparsely supported sectors were therefore interpreted cautiously. Sector‐specific counts are provided in Table S5. Together, these findings indicated a relatively consistent S–SW directional pattern, although the directional results should be interpreted as exploratory surveillance‐derived descriptors because they remain dependent on the selected leading‐edge observations, the spatial reference origin, and the uneven representation of angular sectors. Full results for the spatial origin and leave‐one‐out sensitivity analyses are provided in Tables S4 & S6, respectively.

## 4. Discussion

This study provides a quantitative description of the initial phase of ASF in wild boar in Catalonia by combining surveillance data, a probabilistic reconstruction of mortality timing, and relatively simple metrics of early spatial spread. Three findings are particularly relevant for the outbreak response in newly affected regions. First, enhanced passive surveillance focused on carcass search yielded substantially higher ASFV positivity than active surveillance, further confirming that it is the highest‐yield stream for the early detection and delimitation of the affected area. Second, the probabilistic mortality reconstruction was consistent with ASF‐related deaths occurring before the first detected positive case, indicating that infection may circulate for some time before detection becomes operationally visible. This underscores the importance of robust passive wildlife health surveillance systems for the early detection of ASF outbreaks. Third, the observed epidemic leading edge showed a relatively low apparent mean front‐displacement rate, together with nonlinearity and directional heterogeneity. Importantly, this observed spread pattern should not be interpreted as an intrinsic rate of ASFV spread. It may reflect a combination of underlying epidemic expansion and surveillance and control conditions operating during the study period, including reinforced barriers, systematic carcass removal, and targeted surveillance. Taken together, these findings highlight the importance of sustained carcass‐search capacity, rapid reporting and removal, and the use of early spread descriptors to support adaptive, risk‐based surveillance and zoning.

### 4.1. Passive Surveillance and Detection Yield

The markedly higher positivity under enhanced passive surveillance than under active surveillance indicates a higher surveillance yield within this outbreak dataset and highlights the operational relevance of enhanced passive surveillance, including structured carcass searches using trained dogs, for early ASF detection, delimitation of affected areas, and outbreak monitoring. However, this contrast reflects the different sampling processes and the targeted nature of carcass‐based and event‐based detection and should not be interpreted as a population‐level difference in infection prevalence.

This pattern is consistent with evidence from Latvia showing that the probability of detecting ASFV‐positive wild boar was significantly higher among animals found dead than among hunted animals, supporting the value of enhanced passive surveillance [48]. Lithuanian surveillance data similarly showed substantially higher proportions of ASFV‐positive animals among wild boar found dead than among hunted animals, emphasizing the surveillance value of passive surveillance [49]. At the EU level, European Food Safety Authority (EFSA) epidemiological reports have consistently shown that the proportion of ASFV‐positive animals is much higher among wild boar found dead than among hunted animals and that surveillance of wild boar found dead is central to early detection and outbreak monitoring [10, 35].

Notably, approximately one‐third of the samples classified as enhanced passive surveillance originated from traffic collisions (Table S2), which may partly reflect the dense network of roads, highways, and railways in the affected area. Because traffic collision detections are not specifically targeted toward animals that died from disease, their inclusion may have lowered the aggregate positivity observed for this surveillance stream relative to carcass‐based detections alone.

Beyond empirical comparisons, simulation work indicates that passive surveillance is generally more efficient than active surveillance for early ASF detection in wild boar, although its relative performance depends on carcass recovery, host density, and the epidemic phase [50]. In practical terms, these converging lines of evidence support prioritizing resources for carcass‐based surveillance in the first months of ASFV incursion in a region (reporting channels, rapid sampling logistics, structured search, and safe removal) while recognizing that “passive surveillance” often requires active, organized search to achieve the highest carcass recovery possible.

Not all passive surveillance components were equally informative in this dataset. While whole carcasses and remains found in the field showed the highest ASFV positivity, animals shot on disease suspicion showed intermediate positivity (Table S2). This finding suggests that clinical suspicion or abnormal behavior may enrich sampling for ASFV‐positive animals compared with active surveillance while also indicating that field‐based suspicion has limited specificity, given that ASF can produce severe but variable and often nonspecific, clinical presentations [2, 51]. The estimate for this category should also be interpreted cautiously because it was based on a small number of animals and had a wide CI. Similarly, positivity among animals collected after road or railway collisions was low and only slightly higher than that observed in active surveillance. These data do not support a strong enrichment of ASFV‐positive animals in collision‐derived samples in this outbreak despite the high number of wild boar collisions recorded. Rather, as also observed in previous outbreaks in Europe, the highest surveillance yield appears to come primarily from carcass‐based detection [4, 15].

### 4.2. Hidden Mortality Before First Detection

The probabilistic mortality reconstruction suggests that ASF‐related deaths were occurring before the first carcass was detected, highlighting a recurring challenge in wildlife surveillance: detection dates may lag behind underlying mortality and infection dynamics when carcass discovery is incomplete or delayed. Heterogeneity in time‐to‐detection is biologically and operationally plausible because carcass visibility, decomposition, and access vary strongly in space and time. Field forensics work shows that decomposition and postmortem interval estimation in wild boar vary substantially by microhabitat conditions (e.g., sun vs. shade vs. wallow), which limits precision when inferring death timing from carcass condition alone [52].

These timing uncertainties matter because carcasses are not only surveillance signals but also potential sources of infectious material that may contribute to carcass‐mediated transmission and environmental persistence of ASFV. An accurate estimation of wild boar death dates can help reconstruct the beginning of the outbreak and guide efforts to locate undetected carcasses. Experimental work demonstrates that ASFV can remain stable in carcasses and tissues under environmental conditions, favoring persistence and onward transmission risk and supporting carcass removal as a key component of control [53]. The modeling work further indicates that carcass‐mediated transmission can contribute to ASFV persistence and that interventions affecting carcass detection/removal can change epidemic dynamics [54]. In this context, the long tail of detection delay is operationally important because it implies that undiscovered carcasses may sustain risk even when recent detections appear sparse.

The sensitivity analysis strengthens the interpretation that ASF‐related mortality had already occurred before the first detected carcass while also emphasizing the uncertainty inherent to death‐date reconstruction from carcass decomposition. The similar results obtained under distributions placing more probability toward earlier, midpoint, or later dates suggest that this conclusion was not an artifact of the uniform within‐interval assumption used in the main reconstruction. However, the reconstructed proportion should be interpreted as an approximate surveillance‐based estimate among detected ASFV‐positive carcasses, not as a precise estimate of true cumulative mortality in the wild boar population or of the exact timing of virus introduction.

### 4.3. Front Displacement in Context

Using leading‐edge vertices of cumulative MCPs, the apparent mean radial front‐displacement rate estimated for the Catalan outbreak was 0.042 km/day (95% CI 0.029–0.055), corresponding to ~15 km/year. This estimate is consistent with that of other local ASF outbreaks. For example, Gervasi et al. [36] estimated a directional ASF spread of 33–90 m/day during the early outbreak in northern Italy using a regression approach based on leading‐edge carcasses detected through passive surveillance. Although both studies describe the advancing epidemic front, our approach reconstructs the probable dates of ASF‐related mortality rather than relying on carcass notification dates. This distinction is particularly relevant for ASF because infected carcasses may remain detectable in the environment for weeks or even months, resulting in variable delays between death and detection that can distort the estimates of the epidemic spread.

Comparable local estimates have also been reported using other approaches. Podgorski and Smietanka [55] estimated ~1.5 km/month (≈0.05 km/day) in Poland, while Lentz et al. [56] obtained an initial linear estimate of 0.042 km/day for a German outbreak cluster. An EFSA network analysis estimated median natural propagation velocities of 2.9–11.7 km/year across seven European countries [57]. In contrast, regional or multinational front‐wave analyses can yield substantially higher apparent velocities because they cover broader spatial and temporal domains and may integrate local contiguous spread, heterogeneous case ascertainment, and occasional long‐distance introductions [58].

The relatively low apparent displacement observed in Catalonia is consistent with other estimates from local ASF outbreaks [36, 55, 57] and is compatible with a geographically constrained early outbreak focus. However, this estimate should not be interpreted as a direct measure of ASFV transmission speed, wild boar movement, or an intrinsic epidemiological parameter. Rather, it represents the observed surveillance‐derived leading‐edge displacement under the surveillance and control conditions operating during the outbreak. The observed leading edge likely reflects a combination of local ASF epidemiology, landscape structure, transport and fencing barriers, restrictions on wild boar and human movement, carcass removal, and the evolving surveillance process. Because surveillance intensity and control measures changed throughout the study period, their individual contributions cannot be disentangled. Consequently, the estimated front‐displacement rate integrates multiple processes that could not be separated using the available observational data, including spatial expansion of infection, wild boar mortality, carcass persistence and detectability, reporting delays, landscape accessibility, heterogeneous search effort, and the progressive implementation of control measures. Accordingly, whether an ASFV‐positive wild boar contributed to the reconstructed leading edge depended not only on where and when it died but also on whether, when, and where its carcass was detected.

The definition of the spatial reference origin was another source of uncertainty for the reconstructed leading‐edge metrics. Sensitivity analyses showed that the overall linear apparent front‐displacement rate was relatively stable across plausible origin definitions and leave‐one‐out iterations. The final MCP extent and maximum radial displacement were also relatively stable when individual leading‐edge observations were omitted, whereas segmented regression estimates, particularly the postbreakpoint rate, were more sensitive to both the spatial‐origin definition and the omission of individual observations. This reinforces the interpretation of the segmented results as descriptive features of the surveillance‐derived leading edge rather than mechanistic estimates of the ASFV transmission speed. Directional predictions were more consistent across both sensitivity analyses, with the fastest predicted direction remaining within the S–SW sector; however, these results should still be interpreted as exploratory because they depend on a limited set of leading‐edge detections, the selected spatial reference origin, and the heterogeneous surveillance effort.

#### 4.3.1. Nonlinearity and Apparent Acceleration

Both flexible and segmented models indicated a nonlinear distance–time relationship, with evidence of a change in slope later in the observation period. Although temporal variation in the underlying epidemic expansion is epidemiologically plausible, the observed change may also reflect temporal and spatial variation in carcass detection and search effort. Carcass searches were intensified after outbreak confirmation and were progressively adapted as newly affected areas were identified. This evolving surveillance process could have increased the probability of detecting ASFV‐positive carcasses at greater distances from the defined spatial reference origin.

More generally, at broader spatial and temporal scales, observed ASF expansion may reflect a combination of local contiguous spread, heterogeneous detection, and occasional longer‐distance introductions, which can generate temporal changes in apparent front‐displacement patterns [59]. In addition, modeling work indicates that the timing and intensity of interventions, including carcass detection and removal as well as hunting‐based measures, can alter outbreak trajectories and the feasibility of control, reinforcing that opportunities for early containment may be time‐limited [54, 60, 61]. Because surveillance intensity and control measures changed concurrently, their respective contributions to the observed distance–time pattern could not be disentangled.

The segmented analysis was based on 43 leading‐edge observations. Across the spatial origin and leave‐one‐out sensitivity analyses, the segmented regression parameters were more variable than the overall linear estimate. Although the postbreakpoint rate remained higher than the prebreakpoint rate in every leave‐one‐out iteration, the estimated timing and magnitude of the apparent acceleration were sensitive to individual observations. The breakpoint should therefore be interpreted as a descriptive feature of the surveillance‐derived trajectory rather than as evidence of a mechanistic transition in ASFV transmission or wild‐boar‐mediated spread. Operationally, these considerations support maintaining enhanced passive surveillance beyond the initial delimitation phase because apparent acceleration may reflect either a true change in epidemic expansion or improved detection in newly affected areas.

#### 4.3.2. Directional Heterogeneity and Targeting

Directional analyses indicated heterogeneity in the apparent spread, with higher estimated displacement rates in the southern sector (notably S–SW), although some directions were masked due to limited leading‐edge support. In the Catalan context, this anisotropy may reflect differences in habitat continuity and effective wild boar–habitat connectivity around the initial focus near Cerdanyola del Vallès. Ecological corridors, particularly watercourses and drainage structures allowing wild boar to cross highway barriers toward more continuous forest habitats (e.g., Collserola NP), potentially facilitate southward displacement, while more urbanized areas and major transport infrastructure to the north and east may reduce connectivity. More generally, landscape structure and anthropogenic factors have been shown to influence ASF occurrence patterns in wild boar, supporting the plausibility that connectivity and human footprint shape where cases emerge and how affected areas expand [62]. Accordingly, the S–SW pattern should be interpreted as exploratory and hypothesis‐generating and as an aid for adaptive surveillance planning, rather than as definitive evidence of intrinsic directional transmission of ASFV or directional epidemic behavior.

These results have direct operational implications: surveillance resources, particularly structured carcass search, can be prioritized in directions where apparent displacement appears fastest, while intensified surveillance and management can be focused on ecological corridors likely to facilitate further expansion. This targeting is increasingly evidence‐based; landscape factors were associated with the probability of finding ASF‐positive carcasses across multiple affected countries [63], and simple directional methods have been proposed to support early decisions when data are still limited [36]. Overall, our findings support directionally targeted carcass search and surveillance as part of the early ASF response in newly affected areas, in line with modeling‐based emergency control concepts that emphasize carcass removal and barriers as key response elements [60].

### 4.4. Practical Implications and Limitations

Overall, the findings from the initial study period, 25 November 2025–23 February 2026, of ASF in Catalonia reinforce the notion that carcass detection and testing constitute a highly informative surveillance stream for early detection, situational awareness, and, later, demonstrating absence of circulation, provided that carcasses are actively sought and promptly tested [10, 64]. Operational implementation of structured carcass search has been described in Mediterranean settings, including protocols specifying search effort, team composition, and collaboration with hunters [65]. During the Catalonia outbreak, trained dogs proved to be essential for finding infected wild boar carcasses and remains.

Several limitations should be considered. First, the outbreak is still ongoing, so spread metrics may evolve as more detections accrue. Second, leading‐edge approaches are sensitive to heterogeneity in the detection effort and carcass detectability, which can bias apparent spread and mask directions with limited support. Third, death interval estimates are uncertain even under standardized postmortem evaluation, and the uniform‐within‐interval assumption is a pragmatic approximation [15]. Finally, test positivity should not be interpreted as population prevalence because sampling is surveillance‐driven and conditioned by response zoning and operational limitations and priorities. Despite these limitations, the metrics reported here provide early quantitative benchmarks that can support adaptive zoning, prioritized carcass search, and directionally targeted surveillance during the initial months of a new ASF incursion.

## 5. Conclusion

This study provides an early quantitative characterization of ASF reemergence in wild boar in Catalonia, Spain. Carcass‐based passive surveillance, including search assisted by trained dogs, showed substantially higher qPCR positivity than active surveillance, supporting its prioritization for early detection, delimitation of the affected areas, outbreak monitoring, and management of infection risk. Accounting for uncertainty in the estimated date of death, the probabilistic mortality reconstruction was consistent with ASF‐related deaths occurring before the first detected carcass, underscoring the need for robust wildlife passive surveillance schemes for early ASFV detection. Leading‐edge analyses indicated a relatively low apparent mean front‐displacement rate during the study period, together with evidence of nonlinearity and exploratory directional heterogeneity, including faster apparent advance towards south–southwest sectors where large forest mountain areas are found. These surveillance‐derived patterns describe the observed leading edge and may reflect both underlying epidemic expansion and heterogeneity in carcass detection and search effort. In this peri‐urban Mediterranean landscape with heterogeneous habitat connectivity and variable carcass detectability, these findings support an adaptive, risk‐based response that prioritizes carcass search and removal and uses emerging spatial patterns to refine surveillance and zoning as the outbreak evolves. More broadly, the analytical framework used here may support the early epidemiological assessment of ASF incursions in other newly affected settings.

## Funding

Xavier F. Aguilar, Carles Vilalta, and Osvaldo Fonseca‐Rodríguez were funded by the Ramón y Cajal Programme (Grants RYC2022‐036927‐I, RYC2021‐033095‐I, and RYC2022‐037029‐I, respectively) from the Spanish Ministry of Science and Innovation (MICINN). IRTA was supported by the CERCA Programme/Generalitat de Catalunya.

## Ethics Statement

This study was based on routine animal health surveillance and outbreak response data collected by the competent veterinary authorities. No experimental procedures were performed on animals for the purposes of this study. All data were analyzed in accordance with the applicable regulations and institutional guidelines.

## Conflicts of Interest

The authors declare no conflicts of interest.

## Supporting Information

Additional supporting information can be found online in the Supporting Information section.

## Supporting information


**Supporting Information** The supporting information contains six supporting tables and three supporting figures. Table S1 describes the postmortem decomposition stages used to estimate plausible dates of death. Table S2 and Figure S1 summarize ASFV qPCR results by sampling method, surveillance category, and operational zone. Table S3 and Figure S3 present sensitivity analyses of mortality reconstruction and cumulative mortality timing under alternative within‐interval death‐date distributions. Table S4 examines the sensitivity of leading‐edge front‐displacement estimates to alternative spatial‐origin definitions. Figure S2 and Table S5 present directional front‐displacement estimates and the corresponding sector counts, and Table S6 reports a leave‐one‐out sensitivity analysis of the MCP‐based leading‐edge reconstruction.

## Data Availability

The data used in this study were obtained from official animal health surveillance and outbreak response records and are not publicly available. Requests for access should be directed to the relevant competent authority. Annotated R scripts and simulated example datasets reproducing the main analytical workflow are provided as Supporting Information. These materials contain no real outbreak records or confidential or sensitive outbreak response information.
