# Supplementary material for: Early African Swine Fever Dynamics in Wild Boar After Reemergence in Catalonia, Spain: Surveillance‐Based Estimates of Initial Spread
Source: Transbound Emerg Dis. 2026 Sep 18;2026:3757757. doi: 10.1155/tbed/3757757 (PMC13586855; doi:10.1155/tbed/3757757)
Supplement: Supplementary file 1 [file TBED-2026-3757757-s001.docx]

**Supplementary material**

**Early African swine fever dynamics in wild boar** **after re-emergence in Catalonia, Spain: surveillance-based estimates of initial spread**

Xavier F. Aguilar ^a,b,c^, Liani Coronado ^a,b,c^, Lola Pailler-García ^a,b,c^, Albert Sanz ^d^, Carlos Sánchez ^d^, Marta Busoms ^d^, Francisco Domenes ^a,b,c^, Àlex Cobos ^a,b,c,e^, Enric Vidal ^a,b,c^, Miquel Nofrarías ^a,b,c^, Guillermo Cantero ^a,b,c^, Carme Rosell ^f,g^, Joana Colomer ^f,g,h^, Francesc Accensi ^a,c,e^, Joaquim Segalés ^a,c,e^, Jordi Argilaguet ^a,b,c^, Natàlia Majó ^a,c,e^, Carles Vilalta ^a,b,c^, Osvaldo Fonseca-Rodríguez ^a,b,c,*^

*^a^ Unitat mixta d’Investigació IRTA-UAB en Sanitat Animal. Centre de Recerca en Sanitat Animal (CReSA). Campus de la Universitat Autònoma de Barcelona (UAB), Bellaterra, 08193, Catalonia, Spain.*

*^b^ IRTA. Programa de Sanitat Animal. Centre de Recerca en Sanitat Animal (CReSA). Campus de la Universitat Autònoma de Barcelona (UAB), Bellaterra, 08193, Catalonia, Spain.*

*^c^ World Organization for Animal Health (WOAH) Collaborating Centre for the Research and Control of Emerging and Re-Emerging Swine Diseases in Europe (IRTA-CReSA), Barcelona, Spain.*

*^d^* *Departament d’Agricultura, Ramaderia, Pesca i Alimentació de la Generalitat de Catalunya, Barcelona, Catalonia, Spain.*

*^e^ Departament de Sanitat i Anatomia Animals, Facultat de Veterinària, Universitat Autònoma de Barcelona (UAB), Campus de la UAB, Bellaterra, 08193, Spain.*

*^f^ MINUARTIA, Wildlife Consultancy, 08011 Barcelona, Spain.*

*^g^ Department of Evolutionary Biology, Ecology and Environmental Sciences, Faculty of Biology, University of Barcelona, 08007 Barcelona, Spain.*

*^h^ IRBio, Institut de Recerca de la Biodiversitat, University of Barcelona, Barcelona, Spain.*

* Correspondence: [osvaldo.fonseca@irta.cat](mailto:osvaldo.fonseca@irta.cat)

**Supplementary Table 1.** Post-mortem decomposition stages and associated field indicators in carcasses used to estimate plausible dates of death, adapted from Chenais et al. (2024).

| **Time** | **Stage** | **Description** | **Other internal indicators** |
| --- | --- | --- | --- |
| **0–1 days** | Fresh | Carcass with no signs of decomposition or strong odor. Probable rigor mortis, eyes transparent or slightly cloudy. Fly eggs may be visible. | Blood and meat bright red |
| **2–6 days** | Early decomposition | Leakage of body fluids, skin discoloration.  Fly larvae present, 3–10 mm (especially around the mouth and anus), sunken eyes. | Darkened blood and brownish meat |
| **7–14 days** | Bloating | Swollen carcass, loss of skin and hair due to decomposition. Hair detaches easily, majority of larvae >10 mm externally, and mouth, skin and mucosa blackened.  *Note: bloating may occur earlier, especially in large carcasses.* *Bloating of the digestive tract may occur within ~24 hours post-mortem and should not be confused with stage that refers to generalized carcass swelling due to gas accumulation in tissues.* | Internal putrefaction, subcutaneous tissue darkened, organs identifiable but darkened. |
| **2–4 weeks** | Post-bloating (active decomposition) | Open abdominal cavity filled with larvae >10 mm. Organs reduced to fluid, body fluids dispersed around, muscle loss due to decomposition. | Dry bone marrow (>3 weeks) |
| **1.5–4 months** | Advanced decomposition | Carcass consumed by insects, bones visible (due to decomposition, not scavenging), remaining skin is dry and mummified or putrefied. No viscera. Beetles present. No larvae. | Dry bone marrow |
| **>5 months** | Dry remains | Only skeletal remains, with little remaining skin, with algae or moss growth on bones. |  |

**Supplementary Table 2.** African Swine Fever Virus qPCR positivity of wild boar cases across sampling methods during the study period.

| **Category** | **Total (n)** | **Positive** | **Negative** | **Positivity (95% CI)** |
| --- | --- | --- | --- | --- |
| Captured (net and cage traps/shot in transects/urban areas posing risk to the public) | 796 | 7 | 789 | 0.9% (0.4–1.8) |
| Carcasses and remains found in the field | 395 | 185 | 210 | 46.8% (42.0–51.8) |
| Collected from collisions (roads/railways) | 254 | 4 | 250 | 1.6% (0.6–4.0) |
| Shot on suspicion (clinical signs or abnormal behavior) | 38 | 5 | 33 | 13.2% (5.8–27.3) |

**Supplementary Figure 1**. ASFV qPCR results across wild boar surveillance categories stratified by operational zone during the study period. Category labels in the plot correspond to those used in Supplementary Table 2: Captured (net and cage traps/shot in transects/urban areas posing risk to the public), Carcasses and remains found in the field, Collected from collisions (roads/railways), and Shot on suspicion (clinical signs).

**Supplementary Figure 2.** GAM-based estimate of directional variation in early ASF apparent front-displacement rate (km/day) as a function of bearing from the defined spatial reference origin. The solid line shows the estimated apparent front-displacement rate (km/day) by bearing from the defined spatial reference origin, and the shaded band indicates the 95% confidence interval. Bearing is expressed in degrees clockwise from north. Grey vertical bars denote angular sectors with limited observational support.

**Supplementary Table 3.** Sensitivity of reconstructed mortality and cumulative mortality timing to alternative within-interval death-date distributions.

| **Scenario** | **Deaths by 25 Nov 2025** | **Proportion of reconstructed mortality**  **25 Nov 2025** | **Date reaching 25%** | **Date reaching 50%** | **Date reaching 75%** | **Date reaching 90%** |
| --- | --- | --- | --- | --- | --- | --- |
| Primary: Uniform | 26 (22–29) | 13.1% (11.1%–14.6%) | 18 Dec 2025 (17 Dec 2025–20 Dec 2025) | 12 Jan 2026 (10 Jan 2026–13 Jan 2026) | 27 Jan 2026 (26 Jan 2026–28 Jan 2026) | 06 Feb 2026 (04 Feb 2026–08 Feb 2026) |
| S1: Triangular, midpoint mode | 27 (24–29) | 13.6% (12.1%–14.6%) | 19 Dec 2025 (18 Dec 2025–20 Dec 2025) | 11 Jan 2026 (10 Jan 2026–13 Jan 2026) | 27 Jan 2026 (26 Jan 2026–28 Jan 2026) | 05 Feb 2026 (04 Feb 2026–07 Feb 2026) |
| S2: Triangular, early mode | 28 (25–30) | 14.1% (12.6%–15.2%) | 18 Dec 2025 (17 Dec 2025–19 Dec 2025) | 11 Jan 2026 (09 Jan 2026–12 Jan 2026) | 26 Jan 2026 (26 Jan 2026–27 Jan 2026) | 05 Feb 2026 (04 Feb 2026–06 Feb 2026) |
| S3: Triangular, late mode | 25 (22–28) | 12.6% (11.1%–14.1%) | 19 Dec 2025 (18 Dec 2025–20 Dec 2025) | 12 Jan 2026 (11 Jan 2026–13 Jan 2026) | 28 Jan 2026 (27 Jan 2026–28 Jan 2026) | 06 Feb 2026 (05 Feb 2026–07 Feb 2026) |
| S4: Beta-PERT, midpoint mode | 27 (24–29) | 13.6% (12.1%–14.6%) | 19 Dec 2025 (18 Dec 2025–20 Dec 2025) | 11 Jan 2026 (10 Jan 2026–13 Jan 2026) | 27 Jan 2026 (26 Jan 2026–28 Jan 2026) | 05 Feb 2026 (04 Feb 2026–06 Feb 2026) |
| S5: Beta-PERT, early mode | 29 (27–30) | 14.6% (13.6%–15.2%) | 18 Dec 2025 (17 Dec 2025–19 Dec 2025) | 10 Jan 2026 (09 Jan 2026–11 Jan 2026) | 26 Jan 2026 (25 Jan 2026–27 Jan 2026) | 04 Feb 2026 (03 Feb 2026–05 Feb 2026) |
| S6: Beta-PERT, late mode | 23 (21–26) | 11.6% (10.6%–13.1%) | 20 Dec 2025 (19 Dec 2025–21 Dec 2025) | 13 Jan 2026 (12 Jan 2026–14 Jan 2026) | 28 Jan 2026 (28 Jan 2026–29 Jan 2026) | 06 Feb 2026 (05 Feb 2026–08 Feb 2026) |
| *Values are medians with 95% simulation intervals in parentheses. Simulation intervals were defined by the 2.5th and 97.5th percentiles of the simulated values. Dates indicate when the specified proportion of the 198 carcasses included in the mortality reconstruction had accumulated in each Monte Carlo iteration.* | | | | | | |

**Supplementary Figure 3.** Sensitivity analysis of reconstructed cumulative ASF mortality under alternative death-date distributions.

Supplementary Table 4. Sensitivity of leading-edge front-displacement estimates to alternative spatial-origin definitions.

| **Scenario** | **Spatial origin definition** | **Linear apparent displacement rate, km/day** | **Breakpoint, days** | **Pre-breakpoint rate, km/day** | **Post-breakpoint rate, km/day** | **Fastest predicted direction** | **Maximum predicted directional rate, km/day** |
| --- | --- | --- | --- | --- | --- | --- | --- |
| Primary | Centroid of six earliest reconstructed deaths | 0.0418 (0.0292–0.0545) | 119.0 (98.4–139.6) | 0.0205 (0.0028–0.0382) | 0.1063 (0.0446–0.1679) | S–SW, 213° | 0.0721 |
| S1 | Earliest single reconstructed death | 0.0472 (0.0314–0.0631) | 136.3 (126.4–146.3) | 0.0277 (0.0105–0.0449) | 0.2478 (0.0958–0.3999) | S–SW, 216° | 0.0526 |
| S2 | Centroid of first two detected carcasses | 0.0474 (0.0314–0.0634) | 119.9 (101.0–138.8) | 0.0198 (-0.0023–0.0420) | 0.1346 (0.0574–0.2118) | S–SW, 212° | 0.0448 |
| S3 | Centroid of three earliest reconstructed deaths | 0.0415 (0.0286–0.0544) | 119.0 (96.2–141.8) | 0.0214 (0.0030–0.0399) | 0.1023 (0.0380–0.1666) | S–SW, 216° | 0.0674 |
| S4 | Centroid of five earliest reconstructed deaths | 0.0416 (0.0288–0.0544) | 119.0 (96.9–141.1) | 0.0211 (0.0030–0.0393) | 0.1035 (0.0402–0.1668) | S–SW, 215° | 0.0691 |
| S5 | Centroid of ten earliest reconstructed deaths | 0.0455 (0.0311–0.0599) | 136.5 (126.5–146.4) | 0.0280 (0.0124–0.0437) | 0.2270 (0.0885–0.3655) | S–SW, 210° | 0.0474 |
| *Values in parentheses are 95% confidence intervals. Rates are apparent front-displacement rates derived from surveillance-based leading-edge observations and are not direct estimates of ASFV transmission speed. The temporal origin was kept constant across scenarios to isolate the effect of the spatial-origin definition.* | | | | | | | |

**Supplementary Table 5.** Number of leading-edge observations by directional sector in the primary anisotropy analysis.

| **Directional sector** | **Leading-edge observations** |
| --- | --- |
| N | 5 |
| NE | 0 |
| E | 7 |
| SE | 4 |
| S | 14 |
| SW | 3 |
| W | 3 |
| NW | 7 |

*Directional sectors were defined using 45° angular bins. Counts refer to the 43 leading-edge observations included in the primary anisotropy analysis.*

Supplementary Table 6. Leave-one-out sensitivity analysis of the minimum convex polygon (MCP)-based leading-edge reconstruction.

| **Metric** | **Primary estimate** | **Leave-one-out range** | **Successful iterations** |
| --- | --- | --- | --- |
| Leading-edge observations, n | 43 | 42–48 | 43/43 |
| Final cumulative MCP area, km² | 74.8 | 66.4–74.8 | 43/43 |
| Maximum radial displacement, km | 12.0 | 11.6–12.0 | 43/43 |
| Linear apparent front-displacement rate, km/day | 0.0418 | 0.0396–0.0548 | 43/43 |
| Segmented-regression breakpoint, days | 119.0 | 103.0–154.9 | 43/43 |
| Pre-breakpoint rate, km/day | 0.0205 | 0.0064–0.0367 | 43/43 |
| Post-breakpoint rate, km/day | 0.1063 | 0.0884–0.7863 | 43/43 |
| Fastest predicted direction, degrees | 213 | 195–216 | 43/43 |
| Maximum predicted directional rate, km/day | 0.0721 | 0.0660–0.1166 | 43/43 |
| *Each of the 43 observations classified as leading-edge vertices in the primary analysis was omitted in turn. After each omission, the complete cumulative MCP reconstruction and the linear, segmented, and directional models were repeated while holding the primary spatial and temporal origins constant. Leave-one-out ranges represent the minimum and maximum estimates across iterations and should not be interpreted as 95% confidence intervals. Because the cumulative MCP reconstruction was repeated de novo, previously nonvertex cases could become leading-edge observations; consequently, the reconstructed number of leading-edge observations could exceed 42.* | | | |
